# *Allium sativum* Protease Inhibitor: A Novel Kunitz Trypsin Inhibitor from Garlic Is a New Comrade of the Serpin Family

**DOI:** 10.1371/journal.pone.0165572

**Published:** 2016-11-15

**Authors:** Tooba Naz Shamsi, Romana Parveen, Mohd. Amir, Mohd. Affan Baig, M. Irfan Qureshi, Sher Ali, Sadaf Fatima

**Affiliations:** 1 Department of Biotechnology, Jamia Millia Islamia, New Delhi 110025, India; 2 Centre for Interdisciplinary Research in Basic Sciences, Jamia Millia Islamia, New Delhi 110025, India; Russian Academy of Medical Sciences, RUSSIAN FEDERATION

## Abstract

**Purpose:**

This study was aimed to purify and characterize the Protease inhibitor (PI) from a plant *Allium sativum* (garlic) with strong medicinal properties and to explore its phytodrug potentials.

**Methods:**

*Allium sativum* Protease Inhibitor (ASPI) was purified using ammonium sulphate fractionation and Fast Protein Liquid Chromatography on anion exchanger Hi-Trap DEAE column. The purified protein was analyzed for its purity and molecular weight by SDS PAGE. The confirmation of presence of trypsin inhibiting PI was performed by MALDI TOF-TOF and analyzed by MASCOT database. The ASPI was further investigated for its kinetic properties and stability under extreme conditions of pH, temperature and chemical denaturants. Secondary structure was determined by Circular Dichorism (CD) spectroscopy.

**Results:**

ASPI of ~15 kDa inhibited trypsin and matched "truncated kunitz Trypsin Inhibitor (*Glycine max)"* in MASCOT database. The purified ASPI showed 30376.1371 U/mg specific activity with a fold purity of 159.92 and yield ~93%. ASPI was quite stable in the range of pH 2–12 showing a decline in the activity around pH 4–5 suggesting that the pI value of the protein as ASPI aggregates in this range. ASPI showed stability to a broad range of temperature (10–80°C) but declined beyond 80°C. Further, detergents, oxidizing agents and reducing agents demonstrated change in ASPI activity under varying concentrations. The kinetic analysis revealed sigmoidal relationship of velocity with substrate concentration with Vmax 240.8 (μM/min) and Km value of 0.12 μM. ASPI showed uncompetitive inhibition with a Ki of 0.08±0.01 nM). The Far UV CD depicted 2.0% α -helices and 51% β -sheets at native pH.

**Conclusions:**

To conclude, purified ~15 kDa ASPI exhibited fair stability in wide range of pH and temperature Overall, there was an increase in purification fold with remarkable yield. Chemical modification studies suggested the presence of lysine and tryptophan residues as lead amino acids present in the reactive sites. Therefore, ASPI with trypsin inhibitory property has the potential to be used as a non-cytotoxic clinical agents.

## Introduction

Proteases and their inhibitors are accepted to be predominant in all living entities encompassing microorganisms, plants and animals. Several biological processes such as blood coagulation, hormone processing, complement cascade and apoptosis are conducted by these biological macromolecules [1]. Protease inhibitors (PIs) which develop naturally are elemental for modulating the operation of their corresponding proteases within these pathways [2]. The inhibitors have been grouped in 48 families and graded in four mechanistic classes i.e. cysteine, serine, metallo-protease and aspartic inhibitors which is based on the active amino-acid in their “reaction center” [3].

The utility of protease inhibitors as therapeutic agents, specifically, their purposefulness in inhibition of cellular transformation, blood clotting disorders, osteoporosis, retroviral disease and cancer is under meticulous discovery procedure. One of the most significant biological application of PIs is their capacity to be used as anti-cancer agents by arresting the growth of transformed cells [4–8]. PIs from plant sources have acquired exceptional imporatnce as natural plant protecting agents [9]. PIs, in addition to inhibiting growth of insects and pests, also offer restraining function against pathogenic nematodes like *Globodera tabaccum* [10] and many pathogenic fungi including *Candida tropicalis* [11] and *Trichoderma reesei* [12]. Protease Inhibitors have been decontaminated and defined from a considerable number of plant sources [13–17].

Garlic (*Allium sativum*, Liliaceae) is a acclaimed medicinal plant used for healing and medication and is noted to be a vital culinary spice worldwide. It has been reported since long time for its implicit health benefits and a large number of chemicals from garlic have been isolated demonstrating their application in several human diseases [18–20]. During last two decades, it was shown that garlic is endowed with antibiotic, antifungal [21] and antibacterial activities [22]. Current observations have substantiated that garlic may also be used against antiartherosclerosis, hypolipidemic [23–24] and anticarcinogenesis activities [25–26]. Moreover, several studies have shown garlic to be a potent immunomodulator.

In this study, garlic extract was processed from the edible parts of garlic cloves, Seemingly, ASPI was purified for the first time and its biophysical characterization was done to assess its stability. Further, kinetic enquiry and concluded Vmax, Ki and Km values were conducted. It is envisaged that this study would be useful in exploring the potential of ASPI as a phytodrug in the context of a large number of diseases.

## Materials and Methods

### Materials

*Allium sativum* (PUSA- AG 102) commonly known as “garlic” was obtained from IARI, New Delhi. Chemicals; trypsin (bovine pancreatic trypsin), Nα-benzoyl-DL-arginine-p-nitroanilide (BAPNA), phenylmethylsulfonyl fluoride (PMSF), Polyvinylpyrrolidone (PVP), acrylamide, bis-acrylamide, Tetramethylethylenediamine (TEMED), ammonium persulfate and Sodium Dodecyl Sulfate (SDS), acrylamide, bis-acrylamide, TEMED, ammonium persulfate and SDS were obtained from Sigma-Aldrich. All other reagents and chemicals used were of analytical grade.

### ASPI purification

Garlic bulbs were homogenized in 20mM Tris- 2mM CaCl_2_ (Tris)buffer (pH 8.2) containing 1M NaCl. The homogenate was filtered through muslin cloth and was kept on stirring at 4°C for 4–5hrs. Protease inhibitor such as PVP and PMSF were added to avoid any proteolytic activity. The homogenate was centrifuged at 9000 rpm for 1 hr at 4°C The supernatant obtained was saturated with 30% ammonium sulphate. The supernatant was collected and subjected to 50% ammonium sulphate precipitation and the pellet was obtained after centrifugation at 9000 rpm for 1 hr. This pellet was solubilised in Tris buffer (pH 8.2) followed by extensive dialysis using cellulose tubing (12 kDa cut off) in same buffer for 24 h. The dialyzed sample was filtered and weak anion-exchange chromatography was performed on Hi Trap DEAE FF (1 ml, 7 mm × 25 mm) column (GE Healthcare) pre-equilibrated with Tris buffer (pH 8.2). The sample was injected into the column with a 5-ml loop. The fraction size of eluent and flow rate of buffer were checked by Akta purifier. The unbound proteins were washed till the absorbance at 280 nm dropped down to zero. The bound proteins were eluted with 0-1M NaCl in linear gradient in same buffer. The first peak eluted at 0.12 M NaCl showed trypsin inhibitory activity. The eluent was further concentrated using Amicon filter (Merck, Germany). The protein was then assayed for concentration and activity as mentioned below. The purified protein thus served as ASPI and was characterized further for biochemical assays.

### Protein estimation

ASPI concentration was measured following the protocol of Lowry *et al*. with minor modifications [27] using Bovine Serum Albumin (1mg/ml) as standard.

### SDS-PAGE analysis

SDS-PAGE was performed by the method of Laemmli [28] using 12% resolving gel and 5% stacking gel. The samples were mixed with equal volume of loading buffer and heat denatured before loading into the wells. The proteins were separated at 100V using Mini Protean II unit (Bio-Rad, USA). After complete resolution, the gel was taken off the cast followed by staining in Coomassie Brilliant Blue. Protein standard (10 to 250 kDa) was used for the determination of molecular mass of ASPI. The purity was checked by visualizing the number of bands of the sample run.

### Protein identification by MALDI-TOF-TOF

The protein band from SDS gel was cut and picked in 0.2 ml micro centrifuge tube. Trypsin digestion of excised protein was done by the method of Bagheri et al. [29]. The peptide mass fingerprinting was performed on a MALDI-TOF-TOF MS analyzer (ABSCIEX TOF/TOF 5800, Applied Biosystems, USA) and the protein identification (ID) was made using result-dependent analysis (RDA) by ProteinPilot™ software (Version 3.2, USA). Data was analyzed as MS/MS ion search, and preliminary identification of protein was performed by searching the NCBI database, using the MASCOT (http://www.matrixscience.com) algorithm. To evaluate the protein identification, protein with significant score was considered.

### Trypsin inhibitory activity determination

The trypsin-inhibitory activity of ASPI was determined by measuring the residual enzymatic activity towards the substrate 1.5 mM BAPNA–HCl in 20% Glycerol [30]. The total reaction mixture contained ASPI and trypsin in a ratio of (2:1) and incubated for 15–30 min at room temperature. The chromogenic substrate i.e. BAPNA was added and the reaction mixture was further incubated at room temperature to analyze the reaction of unbound trypsin with BAPNA. The reaction was arrested by adding acetic acid. The enzymatic hydrolysis of BAPNA was determined by visualizing the intensity of yellow color which corresponded to the release of p-nitroaniline and trypsin activity at 410 nm. Activity was defined by the activity of test (PI + trypsin)—Activity of negative (trypsin alone). Activity of trypsin was calculated by the formula:

Activity(U)=△A*Totalvolume(ml)*D.F.*1000000/ξBAPNA*Samplevolume(ml)

Where, ΔA = change in absorbance at 410 nm, D.F. = Dilution Factor, ε_BAPNA_ = Molar extinction coefficient of BAPNA

### Stability studies

#### Effect of pH on ASPI

The effect of pH on the inhibitory potential of ASPI were also checked by pre-incubating it with the enzyme at the desired pH (1–12) for 30 min and then assaying for residual enzyme activity. 100 mM solutions of the following buffers were used to get the desired pH: KCl-HCl (1.0), Gly-HCl (pH 2.0–4.0), sodium acetate (5.0–6.0), sodium phosphate (pH 7.0–8.0) and Gly-NaOH (pH 9.0–12.0). The test sample showing maximum inhibitory activity was taken to be as 100%. Rest of the samples was compared to calculate percentage residual activity.

#### Thermal stability of ASPI

The thermal stability test of ASPI was done in Tris buffer by treating the sample for 30 min at various temperatures, ranging 10–100°C in a thermostat. The samples were then adjusted to 37°C and checked for residual inhibitory activity.

#### Effect of detergents, reducing and oxidizing agents

ASPI was incubated with non-ionic and ionic detergents (Triton X 100, SDS and Tween-80) at 0.5 and l% each w/v for 30 min followed by dialysis against Tris buffer. The residual inhibitory activities were calculated at 410nm by observing chromogenic change after adding trypsin followed by substrate.

The effect of reducing agents β-mercaptoethanol and like Dithiothreitol (DTT) on ASPI activity was analyzed by incubating ASPI with 1, 2 and 3 mmol/L agents for 3 h and their residual inhibitory activities were estimated.

Similarly, the effect of oxidizing agents such as Dimethyl sulphoxide and hydrogen peroxide on ASPI activity was studied by incubating them with ASPI in varied concentrations of 0.5, 1, 2, 3 and 4% (v/v) for 30 min. Later, residual trypsin inhibitory activities were estimated.

### Kinetic analysis

Kineticanalysis of ASPI activity was carried out to calculate Km and Vmax values of ASPI activity. The inhibitory potential of ASPI was analyzed for trypsin using BAPNA (0.005 and0.01 mmol/L). The initial slope “v” was determined for each inhibitory concentration. The velocity of ASPI (V) versus BAPNA concentration (S) was also plotted. The rate of reaction was symbolized as V(OD_410_mM/min/mL). The Vmax and Km values were calculated from the graph. The amidolytic activity of trypsin (30 μg) was determined with various concentrations of BAPNA (1.2 to5.0μM) in the absence of ASPI. The assays were then repeated in the presence of 5 and 15 μg of ASPI. The Ki values were calculated from Lineweaver-Burk plot.

### Structure determination of ASPI

The secondary structure determination of ASPI was determined by Circular Dichorism (CD). Far UV CD spectra were recorded on a J-1500 Jasco spectropolarimeter, equipped with a Peltier-based computer-driven temperature control, and analyzed by means of Jasco software. The cell path was 0.1 cm for measurements in the range of 200–250 nm.

### Statistical analysis

The experiments were done in triplicates. Results were expressed as graphs representing Mean ± SD using the software Graph Pad Prism 5. The results thus confirm the reproducibility of the data.

## Results

### Purification of ASPI

**Fig 1:**
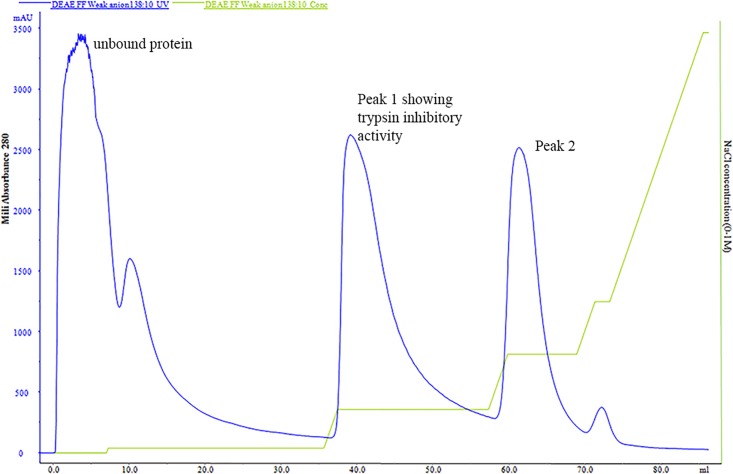
FPLC chromatogram of ASPI after ammonium sulphate saturation and dialysis, the sample was loaded on Hi Trap DEAE FF column and eluted using gradient of NaCl (0–100% in Tris buffer). The chromatogram represents concentration of ASPI eluted (milliabsorbance mA_280_ at 280 nm) on Y axis with amount of eluted fraction (ml) on Y axis. The second curve represents the gradient of NaCl (0–100% of B) where buffer A is 25 mM Tris–HCl (pH 8.2) and B is 1.0 M NaCl in the same buffer.

The purification process yielded ~10 mg of protein from 100 g of peeled fresh garlic bulbs. The sample obtained after ammonium sulphate precipitation and dialysis was subjected to trypsin inhibitory activity. The fractionate thus obtained showed trypsin inhibitory activity and was loaded onto a Hi Trap DEAE FF column. Gradient elution was performed and the fractions were eluted with range of NaCl concentrations (Fig 1). The peaks collected were again assayed for activity against trypsin. The purified ASPI showed 30376.1371 U/mg specific activities with a fold purity of 159.92 and the yield obtained was ~93% (Table 1). The active fraction thus obtained was used further for various biophysical activities.

**Table 1:** Protein Estimation and activity profile at each step of purification.

Step	Activity (U/ml)	Protein Conc.	Amount (ml)	Total Protein (mg)	Total Activity (U)	Specific Activity (U/mg)	Fold Purification	Yield (%)
**Homogenate**	30229.746	1.061	150	159.15	4534461.9	189.945	1	100
**30–50% (NH4)2SO4 precipitate**	164449.82	2.095	26	54.47	4275695.3	3019.0898	15.894	94.293
**FPLC eluent**	301088.27	0.708	14	9.912	4215235.8	30376.137	159.92	92.96

### Protein Estimation

The protein concentrations and trypsin inhibitory activity were determined and compared at each step of purification (Table 1). The results depicted downhill slope of concentration and total protein with each passing step whereas an increase in specific activity and fold purification. The results are interpreted in Table 1.

### SDS-PAGE analysis

**Fig 2:**
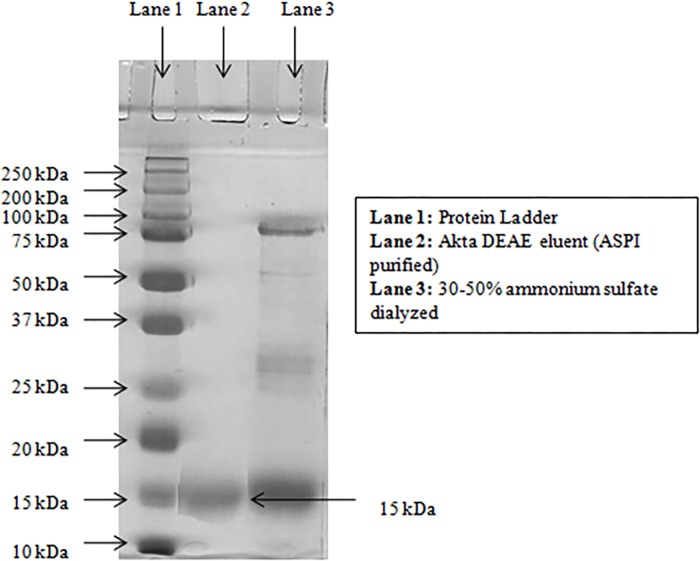
ASPI was run on 12% resolving, 5% stacking SDS PAGE. 20μl of sample with loading buffer was loaded in each well. The bands were visualized using Coomassie staining. Lane 1: standard proteins, Lane 2: Akta-DEAE eluent, Lane 3: 30–50% ammonium sulfate saturated sample (dialyzed).

SDS polyacrylamide gel electrophoresis of the eluted ASPI yielded a single thick band demonstrating purity and molecular weight of the protein. The ASPI showed a clear band corresponding to a molecular weight of approximately ~15 kDa (Fig 2, lane 2).

### Protein identification by MALDI-TOF-MS/MS analysis

The band excised from SDS-PAGE (Fig 2 Lane 2) was identified through MALDI TOF-TOF. A band at ~15-kDa was digested with trypsin into 5 fragments in the range of 997.1549–1762.4725 Da as shown in the peptide mass fingerprint (Table 2). These fragments were found to be the part of Truncated Trypsin Inhibitor (gi|13375351) with maximum mascot score of 92 with the gi|1370187 is the sequence of truncated TI from *Glycine max* with a molecular mass of 16,124 Da and 27% protein sequence coverage. Since the gene sequence of PI from *A*. *sativum* was not determined so far; we considered the purified protein as PI specifically TI of the said plant. Other hits also resembled with the PIs of different plants substantiated the identification of the purified protein. The scoring was based on the “Probability based MOWSE score” i.e. “Ion score” being equal to 10 x Log (P), where P is the probability observed and match was an event occurring randomly. The Peptide sequences obtained after MALDI-TOF-TOF analysis were searched against Swiss-Prot database and MASCOT search which confirmed that the purified protein was trypsin inhibitor.

**Table 2:** List of peptide fragments obtained after tryptic digestion.

S.No.	Mass Mr.	Range	Peptide Sequence
1.	1762.4725	73–88	R.NELDKGIGTIISSPYR.I
2.	1163.3301	78–88	K.GIGTIISSPYR.I
3.	1211.3467	91–101	R.FIAEGHPLSLK.F
4.	1538.2931	132–144	K.IGENKDAMDGWFR.L
5.	997.1549	137–144	K.DAMDGWFR.L

### Trypsin inhibitory activity determination

ASPI was checked for trypsin inhibition using BAPNA as substrate. Activity and Specific activity of PI were calculated at each step of purification. The total activity (activity of protein x total volume of protein) was calculated at each step. The specific trypsin inhibitory activity i.e. total activity of protein/ total amount of protein in fraction increased with each step of purification from ~190 U/mg to 30376.13711 U/mg in 159.15mg homogenate and 9.912 mg FPLC eluent fraction respectively. The purified ASPI showed several fold purification of ~160 compared to crude garlic extract (Table 1).

### Stability studies

#### Effect of temperature and pH on ASPI

**Fig 3:**
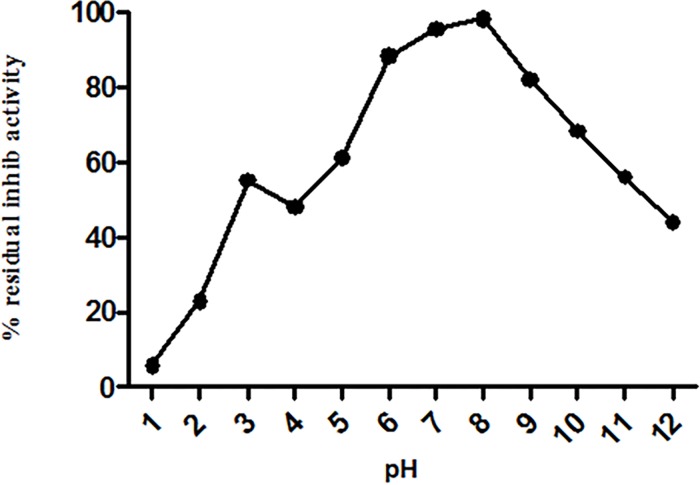
Stability of ASPI was measured at different pH by incubating it in buffers with pH ranging from 0.5–12 for 12 hours.

The ASPI was incubated with buffers of pH 1–12 for 12 hrs at 4°C. Data presented in Fig 3 indicated percentage residual activity of ASPI over a wide range of pH recording maximum drop in activity at pH 4 (~61% activity) compared to the activity observed at other pH. Thus ASPI showed high stability at extreme pH conditions suggesting that ASPI was resistant to pH fluctuations.

**Fig 4:**
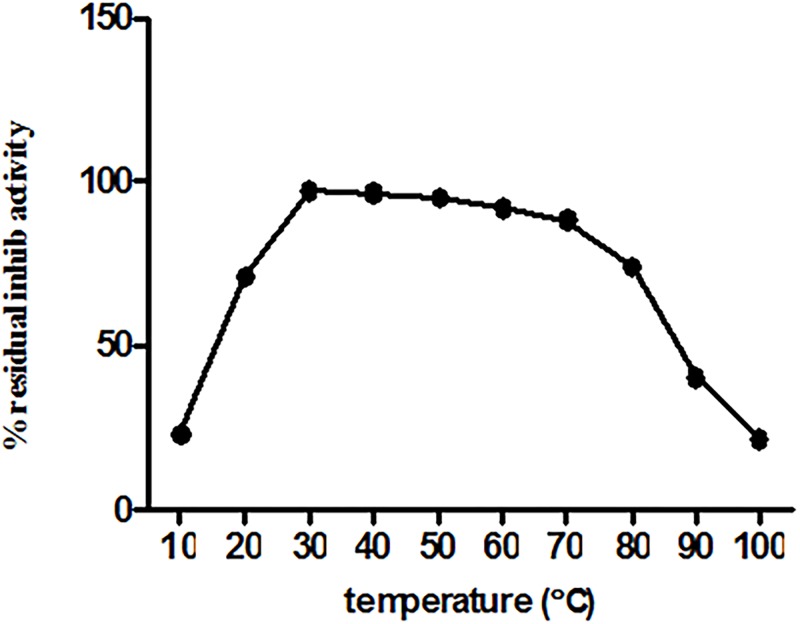
Thermal stability studies of ASPI was done by incubating it at different temperatures ranging from 10–100°C for 30min. Following this, ASPI was assayed for residual inhibitory activity at room temperature.

The residual inhibitory activity of ASPI was measured after pretreatment of ASPI at different temperatures for 30 min. ASPI was found to be resistant to thermal denaturation showing that itretained its activity upto 80°C. The ASPI showed highest activity at the temperatures ranging from 30–40°C (Fig 4). It was noted that activity of ASPI was dropped beyond 80°C and it became totally inactive at 100°C (~21% inhibition). We construe that ASPI is quite heat stable as shown in Fig 4.

#### Effect of detergents, oxidizing and reducing agents on ASPI activity

**Fig 5:**
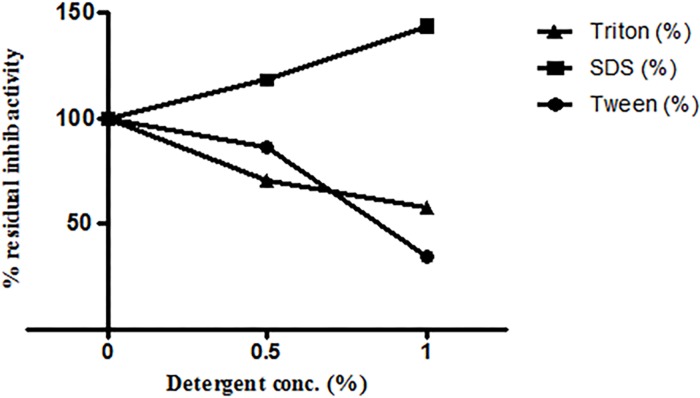
Effect of detergents on inhibitor activity of ASPI by incubating it with detergents for 30 min at room temperature. ASPI was then assayed for residual inhibitory activity at room temperature.

The results depicted that ionic and nonionic detergents except SDS have negative effect on PI activities. There was an increase in residual activity of ASPI to ~143% in the presence of 1% SDS as compared to control. In case of Tween 80 and Triton X 100, the residual inhibitory activities decreased to ~35% and ~58% respectively (Fig 5).

**Fig 6:**
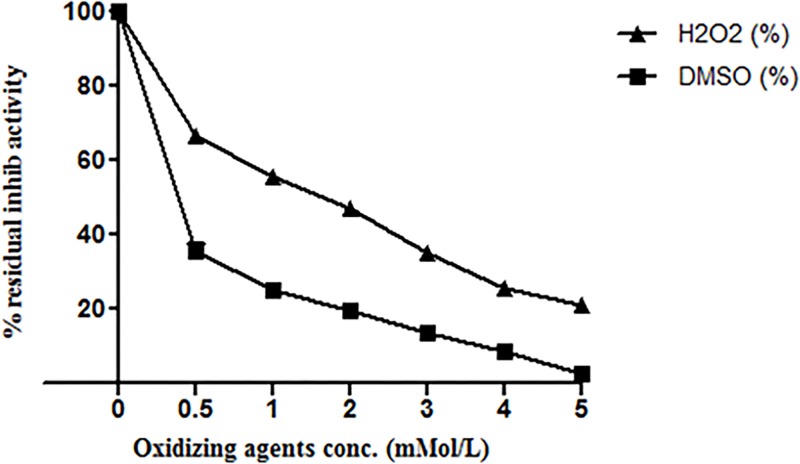
Effect of oxidizing agents on inhibitory activity of ASPI by incubating it with oxidizing agents for 30 min at room temperature. ASPI was then assayed for residual inhibitory activity at room temperature.

Oxidizing agents such as H_2_O_2_ and DMSO reduced ASPI activity in concentration dependent manner. The results showed that 5mM H_2_O_2_ almost completely inhibited ASPI activity whereas 5mM DMSO reduced ASPI activity to ~21% (Fig 6). We can suggest that oxidation can be a key element for ASPI activity regulation.

**Fig 7:**
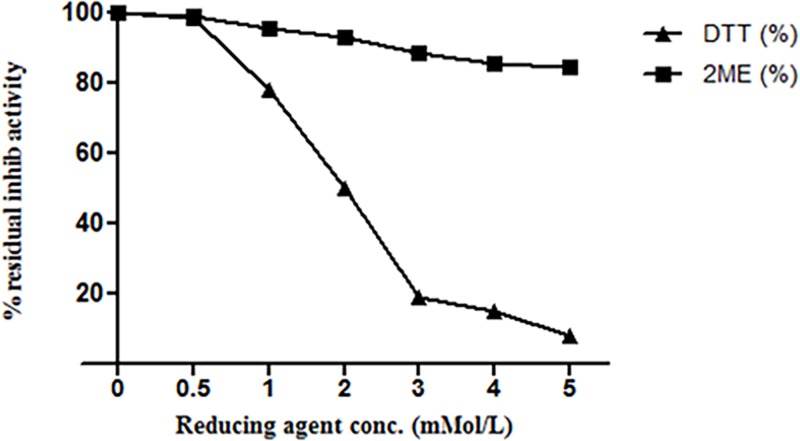
Effect of reducing agents on inhibitor activity of ASPI by incubating ASPI with reducing agents for 30 min at room temperature. ASPI was then assayed for residual inhibitory activity at room temperature.

Moreover, reducing agent like DTT also reduced the residual inhibitory activity of ASPI in concentration dependent manner. The activity was reduced to 8% at 5mM DTT concentration. Whereas, β-ME (1.0–3.0 mM) after 3 hours of incubation barely affected ASPI activity as it remained 99%-93%. Further, an increase in β-ME concentration too could not inhibit ASPI activity to an appreciable level showing maximum drop to ~85% at 5mM β-ME (Fig 7).

### Kinetic studies

**Fig 8:**
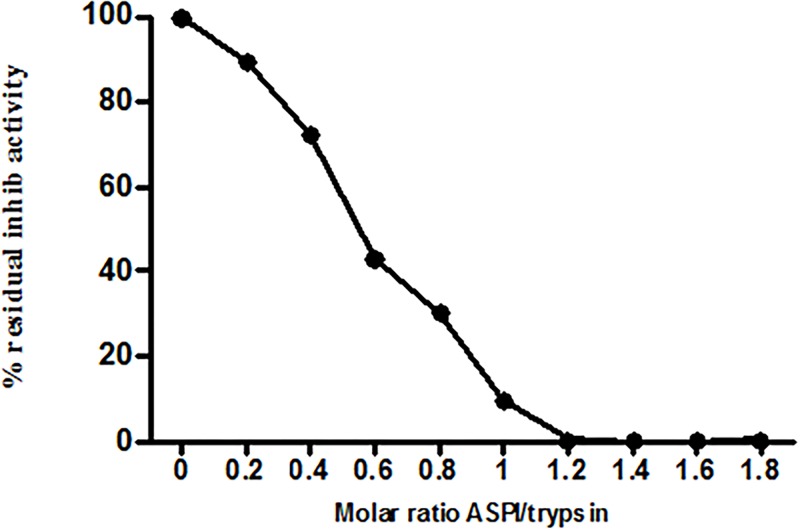
Residual trypsin inhibitory activity in percent with variable function of molar ratio (ASPI: trypsin) using BAPNA at fixed concentration.

**Fig 9:**
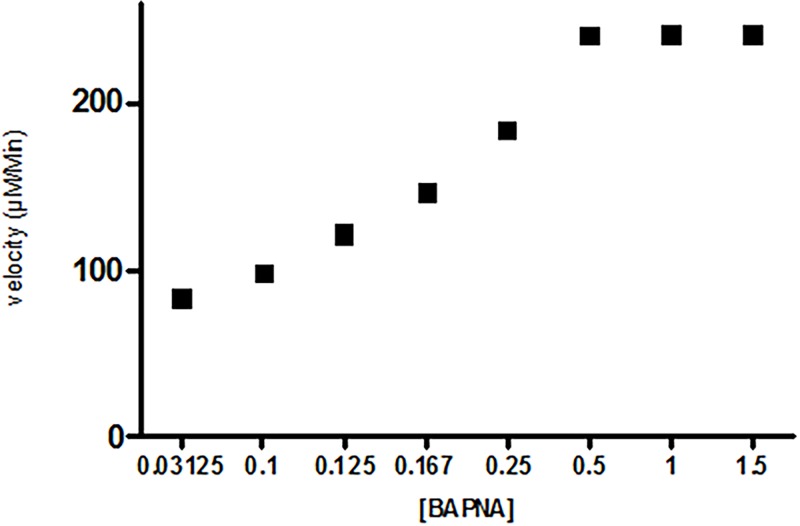
Trypsin inhibitory activity of ASPI showing residual inhibition activity in percent as function of the inhibitor dose at a fixed concentration using BAPNA as variable substrate.

The inhibitory graph with variable ASPI: trypsin concentration with fixed BAPNA volume suggests steep drop in trypsin activity with increasing ASPI/trp ratio. This could be determined spectrophotometrically at 410 nm by observing the decrease in chromogenic substance (Fig 8). The velocity of ASPI was determined by taking variable substrate (BAPNA) concentration. The velocity showed sigmoidal relationship with substrate concentration with the curve meeting, extrapolating X–axis at a point equivalent to -1/km. ASPI showed Vmax 240.8 (μM/min) and Km value of 0.12 μM (Fig 9). The low Km value suggests higher affinity of enzyme (trypsin) towards substrate (BAPNA).

**Fig 10:**
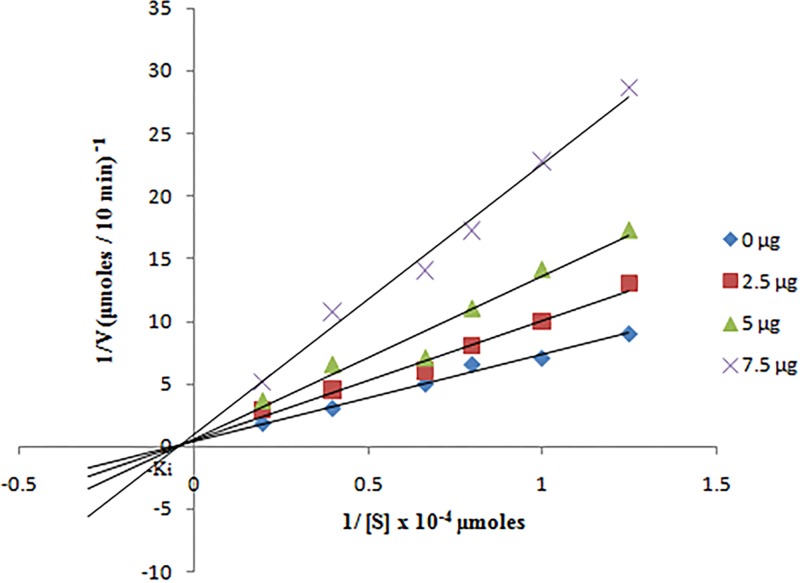
Trypsin inhibition by ASPI was calculated by incubating 30 μg of trypsin with variable ASPI amount (0, 2.5, 5, 7.5 μg) and BAPNA solution (0.8 to 5.0 μM).

Residual trypsin activity in the absence and presence of 2.5 μg, 5 μg and 7.5 μg ASPI was calculated at different substrate (0.8–5 μM BAPNA) concentration. The double reciprocal plot of kinetic data is depicted in Fig 10. In the presence of ASPI, there was a decline in Vmax value and the curves intersecting each other on X axis at -1/km. The mode of trypsin inhibition by ASPI was non-competitive. The Ki value calculated from Dixon plot was 0.08±0.01 nM).

### Structure determination

**Fig 11:**
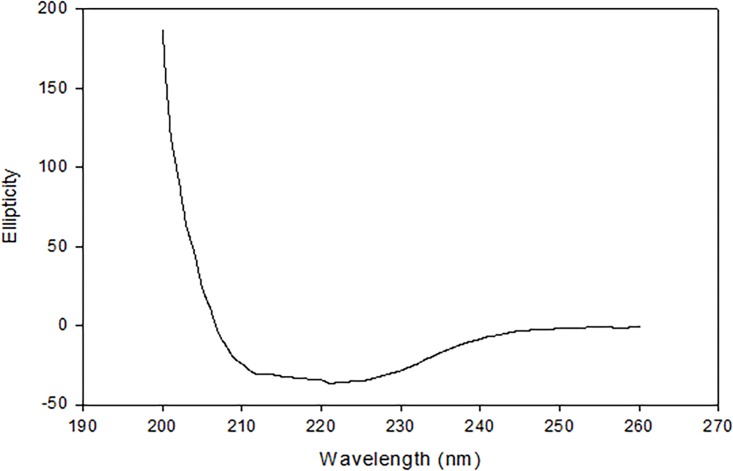
Representative far-UV CD spectra of ASPI in native state at pH 8.2 and 25°C.

ASPI is an α+β protein at pH 8.2, demonstrated by far UV spectrum measured by circular dichroism (Fig 11). Secondary structural content was determined from the CD spectrum of native protein using K2D2 server, which revealed 2.0% α -helices and 51% β -sheets in Tris buffer (20 mM, pH 8.2).

## Discussion

Several plant sources were screened for the presence of PI and the results showed that the samples possess highly stable PI after ammonium sulphate saturation. Selection of *Allium sativum* (garlic a member of the *Liliaceae (Amaryllidaceae)* family) was based on the fact that it has a potent PI with highest medicinal values useful in pharmaceutical, biotechnological and industrial applications. It is known to have lesser amount of protein as compared to legumes [31]. The present work becomes significant and novel because no PI has ever been described from *Allium sativum* so far.

In our study, SDS-PAGE analysis proved that the eluted protein possess a molecular mass of ~15 KDa against the protein standard. Serpins are a superfamily consisting of proteins which inhibit serine protease (trypsin, chymotrypsin etc.) and ASPI being a potent trypsin inhibitor tends to fall in serpin family. Also, the observed molecular mass of the ASPI showed high homology with Kunitz type of inhibitors because members of this family are believed to have molecular mass upto ~16–26 kDa [32–33]. Hence, on the basis of molecular weight of the novel PI we report, it can be inferred that ASPI is a member of family of Kunitz type inhibitor and a member of serpin family. The ability of ASPI to inhibit trypsin proved that it is a strong trypsin inhibitor and being novel. This was further confirmed by MALDI TOF-TOF which matched the database with TI from *Glycine max*.

The results of MALDI TOF-TOF depicted that the protein after tryptic digestion and MALDI analysis matched the sequence of gi|1370187 which is TI from *Glycine max* with a molecular mass of 16,124 Da and 27% protein sequence coverage. ASPI being novel protein does not have its gene sequence available in NCBI database. Thus, the protein purified demonstrating trypsin inhibitory activity is entitled to become ASPI. The results are supported and further strengthened by the resemblance with sequence derived from other TIs of different plants.

Stability of eluted protein was studied in the presence of several chemical and physical denaturants such as pH, temperature and various chemical agents such as detergents, reducing and oxidizing agents. It was assumed that the protein might be functionally stable due to the presence of the intra molecular disulphide bridges in the PI [34]. When the protein was exposed to harsh temperature conditions, it deactivated thermally due to the denaturation and unfolding, because of the disturbance and breakage of covalent and non-covalent interactions [35]. A number of PPIs from plant sources have been purified and found to be highly stable and fairly active up to 70°C [36]. Thermal denaturation of our protein i.e. ASPI at different temperatures (10°C-100°C) depicted that ASPI is a high temperature tolerant protein. The pH stability study of ASPI proved that it was functionally more stable at different pH rangefrom 5.0–10.0 but least stable near pH 4–5 and in highly acidic and alkaline conditions. Owing to pH stability, it may be used as biopesticides. This is, because due to high pH tolerance, it inhibits the highly alkaline serine proteases found in gut flora of insects thereby deactivating the mechanism of digestion of food material and hence kill them [37]. For most of the biotechnological applications, highly thermostable proteins are a must as this boosts the efficiency which is a key requirement for their commercial exploitation [38]. ASPI was also exposed to different concentration of various detergents, oxidizing and reducing agents. Results suggested that purified ASPI was highly active against trypsin even when subjected to high concentration of chemical denaturants such as DTT, beta mercaptoethanol, Triton-X-100, Tween 80 etc. Detergents are commonly involved in protein solubilization from lipid membranes and also sustain protein solubility in solution. An increase in the ASPI activity in the presence of SDS as compared to control suggests that it acts as stabilizer for PI. It has been suggested that oxidation of methionine amino acid found in protein can be one of the reason for high protein activity [39].

The purified ASPI was studied for its kinetics. When a range of variable concentrations of ASPI: trypsin ratio with fixed concentration of substrate BAPNA was used and the inhibitory graph was plotted, it resulted to a steep drop in trypsin activity on raising concentration of ASPI/trypsin ratio. The maximum velocity and the substrate concentration at which the rate of the reaction is suppose to be half of the maximum velocity i.e. Michaelis constant (Km) was also calculated from the sigmoidal curve between velocity vs. substrate concentration. The Vmax and Km value were found to be 240.8 (μM/min) and 0.12 μM respectively. In context to the mechanism of action, ASPI has demonstrated non competitive inhibition which is in compliance to majority of PIs showing non competitive inhibition kinetics [40]. The low Ki value also indicates high affinity of ASPI towards trypsin and also homology with other kunitz-type PIs possessing trypsin inhibitory nature [41].

ASPI being novel protein lacks full protein sequence, therefore, the Multiple Sequence Alignment and homology modeling of ASPI with other TIs could not be performed. However, the structural analysis of ASPI was done by CD measurements which showed majority β-sheets with few α-helix in the secondary structure. Kunitz-type PIs typically possess few α-helix structures and 12 antiparallel β-strands connected by long loops [42]. This confirms ASPI to be a kunitz type serine protease inhibitor. The results obtained in this study suggest that ASPI exhibited fair stability in a wide range of pH and temperature. The high thermal and pH stability of ASPI testifies its applications in various industries including agriculture. In addition, ASPI may further be explored for its clinical and biotechnological applications.
